# Evolution of Antiretroviral Drug Rilpivirine and Approach to Oncology

**DOI:** 10.3390/ijms24032890

**Published:** 2023-02-02

**Authors:** Mariana Pereira, Nuno Vale

**Affiliations:** 1 OncoPharma Research Group, Center for Health Technology and Services Research (CINTESIS), 4200-450 Porto, Portugal; 2 CINTESIS@RISE, Faculty of Medicine, University of Porto, 4200-319 Porto, Portugal; 3 Institute of Biomedical Sciences Abel Salazar (ICBAS), Universidade do Porto (UP), 4050-313 Porto, Portugal; 4 Department of Community Medicine, Health Information and Decision (MEDCIDS), Faculty of Medicine, University of Porto, 4200-450 Porto, Portugal

**Keywords:** rilpivirine, antiretroviral, repurposing, cancer

## Abstract

Rilpivirine is an antiretroviral drug used to treat AIDS worldwide. The drug is a non-nucleoside reverse transcriptase inhibitor that halts the cDNA elongation process and, thus, the capacity of the HIV-1 virus to replicate. With the new wave of drug repurposing in recent years, rilpivirine has been studied in this regard. This drug is useful in Zika virus treatment, with in vivo results indicating regression in neuronal effects often associated with this infection. Several cancer types have also been researched, from breast to leukemia and pancreatic cancer, and rilpivirine has proved to have inhibitory effects in various cell lines with low concentrations, causing cellular death, apoptosis, and cell cycle arrest. The pathways are not yet established, but some works have hypothesized and demonstrated that rilpivirine causes inhibition of Aurora A kinase and has effects on the Janus kinase-signal transducer and activator of transcription (JAK-STAT) signaling pathway and the vascular endothelial growth factors-receptors (VEGFs-VEGFRs) pathway, which are known to be altered in cancer and tumors and can be targeted for cancer treatment. Further testing and clinical trials are needed, but this review demonstrates the potential of rilpivirine’s repurposing for cancer treatment.

## 1. Introduction

According to UNAIDS, 37.7 million people were HIV-positive as of 2020. The immune system is significantly compromised in people with acquired immunodeficiency syndrome (AIDS), which makes the patient more vulnerable to neoplasms and opportunistic infections [1,2]. Since HIV-1 is the most contagious of the two kinds of the human immunodeficiency virus (HIV), it is the primary cause of AIDS worldwide [3].

There are two single-stranded RNA molecules in this virus. The reverse transcriptase (RT), which converts RNA into DNA that may be integrated into the host’s DNA, and the protease, which cleaves protein precursors, are two of the most crucial HIV enzymes and are usually the targets for the treatment of this disease [3]. Nowadays, HIV therapy enables patients to retain immunologic function by reducing viral replication, which contributes to decreasing both mortality rates and transmission rates, with a reduction in sexual transmission of HIV of over 90%. Two nucleoside-analog RT inhibitors (NRTIs) plus either a protease inhibitor (PI), a non-nucleoside RT inhibitor (NNRTIs), or an integrase inhibitor, taken orally daily, make up the standard therapy for HIV infection [4].

Memory CD4+ T cells are the primary cellular target of the HIV virus, which builds up in lymphoid organs and forms viral reservoirs. When infected, memory CD4+ T cells become latently resting and even decline in number. CD8+ T cells, B cells, natural killer cells, and nonlymphoid cells are other cells that are dysregulated by HIV. Together with the previously described loss and dysregulation of CD4+ T cells, this results in a deficit in the immune response to HIV and other infections [5].

## 2. Rilpivirine

**Figure 1:**
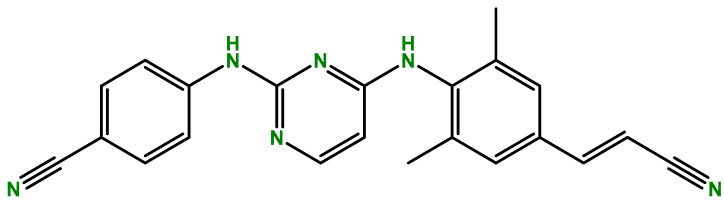
Chemical structure of rilpivirine (developed with ChemBioDraw^®^ Ultra 13.0. A Chemical Drawing Software. Available online: https://chemdrawdirect.perkinelmer.cloud/js/sample/index.html (accessed on 22 December 2022)).

Rilpivirine is a diarylpyrimidine derivative antiretroviral drug, belonging to a group of second-generation NNRTIs and used to treat HIV. This drug is commercially known as Edurant^®^ and is produced by Janssen Pharmaceuticals (Beerse, Belgium), with approval by the US Food and Drug Administration (FDA) and the European Medicines Agency (EMA) in 2011 [6,7]. Figure 1 shows the chemical structure of this drug.

The mechanism of action of NNRTIs involves directly binding with the HIV reverse transcriptase (RT) allosteric site, changing the conformation of the active site. This makes it so that the nucleosides cannot bind to the reverse transcriptase, halting the cDNA elongation process [8]. Since retroviruses like HIV have RNA, the process of reverse transcription of their RNA into cDNA is necessary for its introduction in the host cell genome, an imperative process for viruses, in order to produce key proteins for replication and continuation of infection [9]. Rilpivirine specifically has a cyanovinyl group that positions itself in a hydrophobic cylindrical tunnel of the HIV-RT that connects the NNRTI-binding pocket to the nucleic acid binding cleft, an interaction that is conserved despite RT’s rearrangements, making rilpivirine potent against both wild-type and drug-resistant HIV-1 [10].

Rilpivirine is administered orally through film-coated 25 mg rilpivirine hydrochloride tablets, one tablet once a day after meals, and it is indicated for antiretroviral-treatment-naive patients over 12 years old and with less than 100,000 HIV-1 copies of RNA/mL [6]. It must only be administered after a meal, and it should not be a high-protein meal, since both the fasted state and the high-protein state lead to a decrease of rilpivirine bioavailability of 40% and 50%, respectively [11]. Rilpivirine reaches maximum plasma concentration 4–5 h after oral consumption, with an AUC_24h_ of around 2397 ng.h/mL and a steady-state plasma concentration of around 152 ng/mL, which equalsabout 0.4 μM [12]. Once in the blood, 99.7% binds with plasma proteins, mainly with albumin, which gives a free plasma concentration of around 1.2 nM [7]. Solubility and bioavailability of rilpivirine are also dependent on the existence of an acidic pH of the gastrointestinal tract, which was proven with the concomitant administration with omeprazole that increased gastric pH and led to a decrease of plasma concentration and AUC. Therefore, rilpivirine should not be given with proton pump inhibitors, so as to not decrease its efficacy [13].

**Figure 2:**
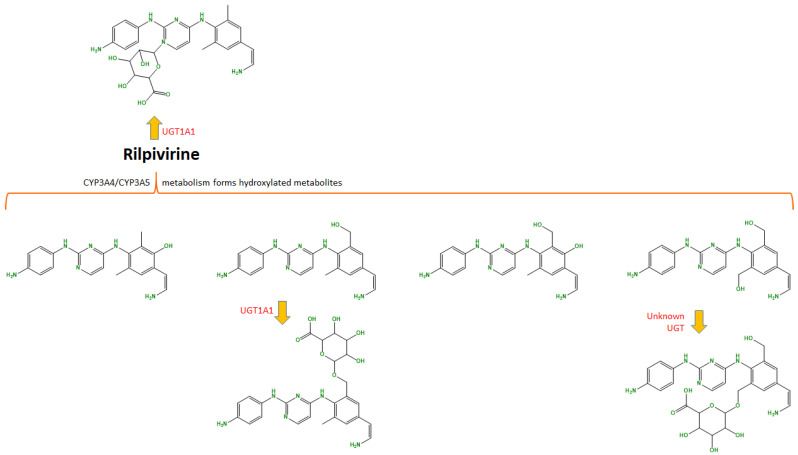
Rilpivirine is administered orally and suffers hepatic metabolism from CYP3A4 and, to a lesser degree, CYP3A5, into several hydroxylated metabolites, and some are then glucuronidated by UGT1A1 and an unknown glucuronidate. It can also be glucuronidated directly by UGT1A1. (Developed with ChemBioDraw^®^ Ultra 13.0. A Chemical Drawing Software. Available online: https://chemdrawdirect.perkinelmer.cloud/js/sample/index.html (accessed on 22 December 2022)).

The main pathways of metabolism involve CYP3A4, and, to a lesser extent, CYP3A5, both of the cytochrome P450 system. It is an oxidative metabolism that creates several hydroxylated metabolites, which then are transformed through UGT1A1 and other glucuronidates into glucuronidated metabolites. To a lower degree, rilpivirine can also be glucuronidated directly via UGT1A4 (Figure 2). The drug has a half-life of around 50 h. The elimination is mostly through feces (85%), of which 25% is the unchanged drug. In urine, which accounts for 6.1% of elimination, less than 1% is the unchanged drug [7,14].

In terms of its interaction potential with other drugs, a study was performed to understand the effect of rilpivirine in transporters (P-glycoprotein P-gp, multidrug resistance proteins MRP1 and MRP2, breast cancer resistance protein BCRP, and organic anion transporting polypeptides OATP1B1 and OATP1B3), both in inhibition and in induction, as well as in drug-metabolizing enzymes, namely CYP3A4, CYP2B6, CYP2D6, and CYP2C19 (also in inhibition and induction) [15]. The results demonstrated that rilpivirine was not transported by any of the transporters. Meanwhile, it proved to have an inhibitory effect in all drug transporters as well as in the cytochrome P450 enzymes, with IC_50_ values ranging from 1.3–13.1 μM. Overall these concentrations are above the normal free rilpivirine concentration in plasma of 1.2 nM [7], therefore there is a low probability of systemic effects. However, they could be relevant in the intestinal tract and the inhibition of these enzymes and transporters could potentially interfere with the absorption and bioavailability of other drugs. Overall, despite interference with transporters and enzymes, rilpivirine has a low chance of interacting with co-administered drugs, accounting for its low free-plasma concentration, similar to its structural analog etravirine, proving that second-generation NNRTIs are less likely to have drug-drug interactions [15].

The combination of rilpivirine and cabotegravir has been of interest in recent years. Cabotegravir is an integrase strand transfer inhibitor (INSTI), one which inhibits the integrase enzyme from integrating viral cDNA into the hosts’ chromatin by processing the 3′ end of the DNA strands, which will then cut the hosts’ DNA and join the 5′ ends. This mechanism is key for virus replication [16]. This drug exists in a short-acting oral formulation but also in a long-acting injectable form, both composed of the drug in nanocrystal form, under the commercial name of Vocabria^®^ produced by ViiV Healthcare (Brentford, United Kingdom), and it has been indicated for pre-exposure prophylaxis [17,18]. A new therapeutic kit was concocted by combining long-release cabotegravir (400 mg) and rilpivirine (600 mg) suspensions for monthly intramuscular injection named Cabenuva^TM^ in a collaboration between Janssen Pharmaceuticals (Beerse, Belgium) and ViiV Healthcare (Brentford, United Kingdom), that can be taken at most, monthly [19]. This co-package was approved by the FDA in 2021 as a complete regimen for virologically suppressed patients without a history of treatment failure, and it is to be preceded by the administration of oral cabotegravir (30 mg) and rilpivirine (25 mg) daily for a month, so as to establish tolerability of the patient [20].

The efficacy of this combination was demonstrated in several clinical trials. Firstly, the oral administration of these two drugs was tested in the Long-Acting Antiretroviral Treatment Enabling (LATTE) 2b trial (NCT01641809). Here, the patients were divided into groups taking cabotegravir (10/30/60 mg) and rilpivirine (25 mg) daily vs groups under the standard antiretroviral therapy (ART) of efavirenz and two NRTIs. At 96 weeks, the dual maintenance regime of cabotegravir and rilpivirine had similar efficacy to the standard ART regime, with the best and safest dose of cabotegravir being 30 mg [21]. A second clinical trial was performed afterward to assess the safety and efficacy of the same combination, but this time in a long-acting injection form, in the LATTE-2 2b study (NCT02120352). Patients with viral suppression were divided into 4- or 8-week intervals of intramuscular long-acting cabotegravir and rilpivirine injections (400/600mg 2 mL and 600/900 mg 3 mL, respectively), or oral cabotegravir with abacavir-lamivudine daily. The results after 96 weeks demonstrated that the long-acting injections were just as effective as the three-drug combination taken daily, while still being well tolerated and without any serious adverse effects related to the drugs [22]. After these initial 96 weeks, this trial continued for 5 years and it demonstrated, beyond the efficacy of long-acting cabotegravir and rilpivirine, the durability of this regimen both monthly and bimonthly, without severe secondary effects even after long use [23]. After these phase two studies, phase three clinical trials were performed and funded by Janssen and ViiV Healthcare to prove the effects of this dual drug combination, namely the First Long-Acting Injectable Regimen trial (FLAIR, NCT02938520), which focused on patients that have never received any form of antiretroviral therapy [24], and the Antiretroviral Therapy as Long-Acting Suppression trial (ATLAS, NCT02951052), that tested patients already doing a regimen, and that were in viral remission [25]. Both 48-week studies demonstrated that the monthly long-acting rilpivirine and cabotegravir injections were non-inferior to oral therapy with standard drugs, in patients both treatment-naïve and that already had previous antiretroviral regimens, which was what eventually led to the approval of this new regimen by regulatory entities [24,25]. An ATLAS-2M phase 3b clinical trial has also demonstrated similar safety and efficacy of the 8-week interval dosing (600/900 mg) in comparison with the 4-week interval (400/600 mg) [26], which has led to the approval of a two-month regimen of this combination in Europe as well [27].

## 3. Repurposing of Rilpivirine

Drug repurposing is a rising technique in the search for new disease treatment methods, in which preexisting drugs for a determined illness are used in a different one entirely, with the main area of investigation being cancer. This helps bypass the high costs and slow pace that are inherently associated with the development of new drugs, since the repurposed ones already have gone through the steps needed to assess their risk and are considered safe for human use [28].

The logical first step in the repurposing of rilpivirine is for other viral diseases. The Zika virus is a widespread pathogen of rapid transmission that causes neurological disorders. This virus has an RNA genome, with a positive RNA strand that codes for the NS5 protein, crucial for the replication of the virus [29]. A study tried to understand the effect of rilpivirine on brain models infected with the Zika virus. In vitro studies were initially performed using several types of NRTIs and NNRTIs in human fetal and adult astrocytes infected with the Zika virus at concentrations of 5 and 25 μM for 72 h. Rilpivirine-only had a significant effect on viral suppression in both cell types. The study of protein levels showed that rilpivirine had a concentration-dependent inhibitory effect on NS1, NS3, and NS5 proteins. After these results were obtained, in silico studies focused on how rilpivirine specifically inhibited NS5, by using docking models of various binding sites of this protein. Rilpivirine is likely to bind to an RNA-dependent RNA polymerase (RdRp) domain on the C terminal of the NS5 protein, a prediction that was then corroborated with protein assays. Further in vivo testing was performed using a Zika-virus-infected interferon-alpha/beta receptor (IFN-A/R) knockout mouse model treated with 12.5 μg of rilpivirine per 25 g mouse, which were sacrificed 14 days after infection. Rilpivirine exposure led to a decrease in mortality, since all infected mice treated with this antiretroviral survived the 14 days of the study, while several infected non-treated did not. After sacrificing the mice and analyzing the different organs, it was noted that rilpivirine treatment suppressed viral replication, and protected the brain and neurons from damage, since while there was still some inflammation present, there was no cell damage related to necrosis and apoptosis, unlike in the control group. The inhibition of NS5 activity was also validated in vivo. Altogether, these are interesting results that indicate that the repurposing of rilpivirine, and possibly other NNRTIs, is a potential therapeutic approach for the prevention and treatment of Zika virus-related sickness [30].

Another group of diseases that are often a subject of repurposing studies are cancers and similar neoplasia. Given this, the repurposing of rilpivirine for cancer will be subsequently discussed.

Conducting web research of studies using rilpivirine in cancer models yielded no results using models of either bladder or prostate cancer, nor did it yield results of works using rilpivirine in combination with other drugs.

We only found a few studies using rilpivirine in cancer models. The first study developed phenylamino-phenoxy-quinoline derivatives from overlaying the structures of rilpivirine and two other NNRTIs, nevirapine and etravirine, and tested the parent drugs and the new derivatives in cancer cell lines. For this review, the results of interest are the ones obtained for rilpivirine alone. This compound was tested at various concentrations in several cancer cell lines, namely HepG2 hepatocarcinoma, MOLT-3 acute lymphoblastic leukemia, HuCCA-1 cholangiocarcinoma, A549 lung carcinoma, MDA-MB-231 hormone-independent breast cancer, S102 Thai liver cancer, HeLa cervical carcinoma, T47-D hormone-dependent breast cancer, H69AR multidrug-resistant lung cancer, and HL-60 promyeloblast cell lines, all for 48 h. The best results for IC_50_ were obtained for MOLT-3 acute leukemia (4.3 ± 0.5 μM), HeLa cervical cancer cells (11.3 ± 1.2 μM), HL-60 promyeloblast (11.5 ± 0.8 μM), and T47-D hormone-dependent breast cancer (15.0 ± 1.3 μM). Meanwhile, rilpivirine showed no effect in A549 lung carcinoma cells, but it did show effects in the other lung carcinoma line (H69AR), although only at high concentrations (57.1 ± 2.0 μM). In all the other cell lines, the drug had an effect, with IC_50_ values ranging from 22.5–87.4 μM. This study demonstrated that rilpivirine has antineoplastic activity in several cell lines associated with multiple types of cancer, making this drug an interesting candidate for drug repurposing [31].

Another work studied several clinically-used NNRTIs in pancreatic cell models (BxPC-3 and Panc-1) for 72 h. Rilpivirine, together with efavirenz, proved to be the most cytotoxic at low concentrations in BxPC-3 cells, with rilpivirine having a 50% effective concentration (EC_50_) of 24.4 µM and 16.2 µM, obtained from apoptosis and colony-forming assays, respectively. This drug has a slowly-increasing toxicity proportional to the increase of concentration and also had toxicity on Panc-1 cells, but with a higher EC_50_ (294 µM). The toxicity at lower concentrations is associated with apoptosis, while for higher concentrations, necrosis appears to be the leading cell death pathway. The blood levels of rilpivirine in patients were also tested; rilpivirine had a mean level of 0.39 µM, ranging from 0–1.56 µM, which is considerably lower than the values needed for the promotion of apoptosis in pancreatic cancer cells [32]. However, this could be bypassed by direct intravenous administration of rilpivirine, which would increase blood levels, since it would not experience the first passage effect and low absorption values, or its combination with anti-cancer drugs, which could help to potentiate the effect, even with lower blood concentrations.

Another recent work developed an extensive study of, among other drugs, rilpivirine in aurora kinase inhibition and cancer cell growth arrest, using in silico and in vitro methods [33]. The aurora family is composed of three serine/threonine kinases (A/B/C) that have catalytic domains with phosphorylating sites. Aurora A and B are expressed in many cell types and are related to cell-cycle progression, from G2 through cytokinesis, while aurora C is mostly associated with meiosis [34]. Aurora A specifically is associated with centrosome maturation, entrance in mitosis, and mitotic spindle formation, being degraded to low levels after mitosis [35]. It has been demonstrated, however, that there is an overexpression of this kinase in various types of hematological and solid cancers, one which is associated with poor prognosis and survival. The promotion of tumorigenesis is related to an increase in proliferation through cell cycle entry induction, and a decrease of apoptosis, with genomic and chromosomal instabilities, due to inactivated DNA damage checkpoints [36]. Because of this, aurora A is a target for inhibition in cancer therapeutics. The study proposed to find from a database with thousands of drugs a possible repurposed drug that could inhibit aurora A. The initial screening was performed through in silico methods. The drugs were tested for fitting in the binding site of the target and compared with a ligand of aurora A, among other requirements. A total of 24 drugs were selected; these were then tested for growth inhibition in hematological (acute myeloid leukemia) and solid tumor (colorectal carcinoma) cell lines at concentrations of 25, 50, and 100 µM. Four drugs presented an inhibition greater than 70% at the lowest concentration, in which rilpivirine, and its NNRTI analog etravirine, were included. Afterward, the half-maximal growth inhibition of the drugs was assessed in cell lines of breast, colon, leukemia, ovarian, and pancreatic cancers at 72 h. Rilpivirine showed values between 3.045–9.422 µM, and this, combined with its structural similarity with the ligand and extensive pharmacokinetic data, led to the further focus on rilpivirine in this study. The drug had a potent inhibitory effect on aurora A kinase (>99%), with a constant of inhibition at 0.116 µM, and a decrease in autophosphorylation of aurora A at the Thr288 site was noted in breast cancer T47D cells exposed to rilpivirine. This is the common pathway through which aurora A kinase inhibitors usually operate [37]. Lastly, they also showed that rilpivirine arrests the cell cycle at the G2/M phase and induces apoptosis on T47D cells, both in concentration and time-dependent manners. Taken together, these results show that rilpivirine has a proven anti-cancer effect related to aurora A kinase inhibition in breast cancer cells, which are interesting results for the possible use of rilpivirine in cancer treatment [33].

The works mentioned above show that rilpivirine can cause an anti-cancer effect, even at relatively low concentrations, and are summarized in Table 1. Despite the effect’s having types different from what we will want to study, this demonstrates the potential of this drug in anti-cancer treatment, which is why it is important to take the research of Rilpivirine further, particularly in lung, bladder, and prostate cancer, which are amongst the most lethal cancer types.

## 4. Possible Anti-Cancer Pathways of Rilpivirine

**Figure 3:**
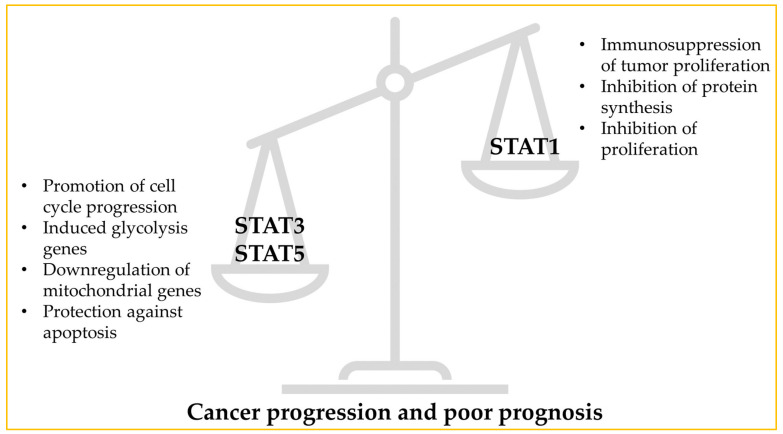
Effects of STAT1/3/5 in cancer progression and poor prognosis of patients.

The Janus kinase-signal transducer and activator of transcription (JAK-STAT) signaling pathway is an important communication pathway in cells, with several constituents and effects in various organism events, such as hematopoiesis and inflammation [38]. JAKs are tyrosine kinases that are associated with cytokine receptors and they transactivate by phosphorylation of tyrosine residues in the cytoplasmatic side of the receptors. This creates a docking site for STATs, which are then phosphorylated and form dimers that move to the nucleus and bind with promoter sites, activating or repressing gene transcription [39]. STAT3 and STAT5A/B have actually been associated with the promotion of oncogenesis. STAT3 potentiates cell cycle progression through the decrease of inhibitors, such as p21, and the increase of promoters, like cyclin-dependent kinases. Meanwhile, STAT5 has an anti-apoptotic effect, through the activation of the production of Bcl-xL, an anti-apoptotic protein [40]. Tumor cells are also characterized by the transition from oxidative phosphorylation to glycolysis, something that STAT3 induces by increasing the transcription of glycolysis genes and downregulating mitochondrial genes [41]. Meanwhile, STAT1 has been known to have a tumor-suppressing effect by promoting immunosurveillance of tumors and having effects on cell-autonomous mechanisms, inhibiting protein synthesis and cell proliferation [42]. Overall, this pathway seems to be of interest in cancer treatment, as it has been shown that patients of several cancer types, such as lung and prostate cancers, have a worse outcome when STAT3 and 5 are activated, while STAT1 activation is generally associated with better prognosis [40]. Figure 3 shows a resumed representation of the STAT genes’ effects on cancer progression and prognosis.

The effect of rilpivirine in the JAK-STAT pathway was tested by using models of liver fibrosis [43]. This is a serious disease that can lead to liver dysfunction and cause hepatocarcinoma, for which there is no cure yet [44]. Hepatocytes and hepatic stellate cells (HSC), which, when activated, contribute to the progression of this disease, were exposed to clinically relevant concentrations of 1–8 μM of rilpivirine for 24–72 h. The results obtained demonstrated that there was an increase of STAT1 signaling in HSC cells, with a pro-apoptotic effect that was not observed in hepatocytes. Conversely, the levels of STAT3 in normal hepatocytes increased, which led to the regeneration of hepatocytes, an effect that was not only secondary to the STAT1 increase in HSC cells but also a consequence of the increased apoptosis of those cells. This study demonstrates that rilpivirine is capable of interfering with the JAK-STAT pathway in a way that degenerates diseased cells while improving the condition and number of healthy cells. In this work, a clinical study of the hepatic function of HIV patients that have anti-HIV regimens with vs. without rilpivirine was also performed, and it demonstrated that patients that take rilpivirine have an overall better liver function [43]. Taken together, there is a clear potential for the use of rilpivirine to interfere with the JAK-STAT pathway and, while the evidence was with liver fibrosis, there could be similar effects on hepatocarcinoma, warranting further investigation.

The vascular endothelial growth factors-receptors (VEGFs-VEGFRs) pathway is key in the development and maintenance of new blood vessel structures at several stages in life. One of these receptors, the VEGFR-2, a tyrosine kinase receptor, has a particular effect on angiogenesis [45]. This process is related to the formation of new blood vessels by tumors from pre-existing vessels, and these are typically abnormal and occur by increasing permeability of the pre-existing vessels so that the endothelial cells and other components can pass and form these new vessels. An angiogenesis switch is often associated with tumor promotion, as well as the progression of pre-malignant lesions to invasive and metastatic cancer [46]. VEGFR-2, when autophosphorylated due to ligands, activates a downstream pathway that leads to a pro-angiogenic effect, mainly associated with cellular responses in endothelial cells, like the promotion of cell division and survival [47]. This receptor is, therefore, a target for inhibition in cancer therapy, and several drugs already exist for this purpose and have found great success, but they often have deleterious side effects for the patients [48]. Thus, an investigation group set out to discover, through an in silico approach, if any pre-existing drugs could potentially inhibit VEGFR2. The implementation of this workflow combined the information obtained from known VEGFR-2 inhibitors, modeling, and molecular docking to test 1841 FDA-approved drugs for possible VEGFR-2 inhibition. Of these, nine had interesting results and were chosen for in vitro testing of receptor inhibition, one of which was rilpivirine. This drug demonstrated an IC_50_ of 5.45 ± 1.10 µM, only slightly higher than that of the reference drug used axitinib (2.82 ± 0.43 µM), with a novel scaffold against VEGFR-2. This demonstrated that rilpivirine has inhibitory activity against this receptor and is promising for antiangiogenesis and tumor treatment [49].

## 5. Conclusions

Rilpivirine is an antiretroviral drug widely used, acting as a reverse transcriptase inhibitor. The repurposing of this drug has been discussed in this review. The potential has been demonstrated for Zika-virus infection-related effects, with viral repression and neurological protection. The drug has also shown effects in cancer cell models, from breast to pancreatic and leukemia cells, causing cell death through apoptosis and cell cycle arrest. Pathways of antineoplastic effects have been hypothesized, namely inhibition of aurora A kinase and interactions with the JAK-STAT and the VEGFs-VEGFRs pathways, mainly through inhibition of VEGFR-2. Overall, this review demonstrated the potential of rilpivirine’s repurposing, for virus infections as well as for cancer treatment, even if more testing for pathway discovery and in vivo and clinical results are needed.

## Figures and Tables

**Table 1:** Works related to the repurposing of Rilpivirine in cancer.

Cancer Type	Model	Results	Ref
Lymphoblastic leukemia	MOLT-3 cells	Antineoplastic effect of rilpivirine in cancer cells with IC_50_ ranging from 4.3–57.1 μM at 48 h	[31]
Cervical cancer	HeLa cells		
Promyeloblast	HL-60 cells		
Breast cancer	T47-D cells		
Lung carcinoma	H69AR cells		
Pancreatic cancer	BxPC-3 and Panc-1 cells	Cytotoxic at low concentrations in BxPC-3 cells, with the promotion of adverse effects in colony formation at 16.2 μM and apoptosis at 24.4 μM at 72 h. Cytotoxicity in Panc-1 cells at 294 μM at 72 h	[32]
Acute myeloid leukemia	Cell lines	IC_50_ values between 3.045–9.422 µM in cell lines tested at 72 h. Inhibitory effect in aurora A kinase at 0.116 µM	[33]
Colorectal carcinoma			
Pancreatic cancer			
Ovarian cancer			
Breast cancer	T47-D cells	Decreased autophosphorylation of Aurora A, leading to its inhibition. Cell cycle arrest at the G2/M stage and induced apoptosis in a time and concentration-dependent manner	

## Data Availability

Not applicable.
